# Identification of signaling pathways associated with achaete-scute homolog 1 in glioblastomas through ChIP-seq data bioinformatics

**DOI:** 10.3389/fgene.2022.938712

**Published:** 2022-09-06

**Authors:** Na Zhang, Jie Zhang, Zhihong Liu, Tushuai Li

**Affiliations:** ^1^ School of Food and Bioengineering, Xuzhou University of Technology, Jiangsu, Xuzhou, China; ^2^ School of Biology and Food Engineering, Changshu Institute of Technology, Jiangsu, Suzhou, China; ^3^ The State Key Laboratory of Pharmaceutical Biotechnology, Medical School, School of Life Sciences, Nanjing University, Nanjing, Jiangsu, China; ^4^ School of Food Science and Technology, Jiangnan University, Wuxi, Jiangsu, China; ^5^ Wuxi School of Medicine, Jiangnan University, Wuxi, Jiangsu, China

**Keywords:** achaete-scute homolog 1 gene, glioblastomas, bioinformatics, ChIP-seq, signaling pathways

## Abstract

**Background:** Achaete-scute homolog 1 transcription factors were important in the differentiation of neuronal-like glioblastoma (GBM) cancer stem cells (CSCs). To gain a better understanding of the role of ASCL1 in GBM, chromatin immunoprecipitation followed by high-throughput sequencing (ChIP-seq) data can be analyzed to construct their gene transcription regulation network.

**Methods:** GSE87618 was downloaded from the Gene Expression Omnibus, which is a famous database, in the field of biology. The filtered clean reads were mapped to the human genome utilizing the software of bowtie2. Then, differential peak analysis was performed by diffbind. Finally, the annotated gene functions and signaling pathways were investigated by Gene ontology function and kyoto encyclopedia of genes genomes (KEGG) pathway enrichment analysis. Moreover, the protein–protein interaction network (PPI) analysis of genes obtained from ASCL1 was carried out to explore the hub genes influenced by ASCL1.

**Results:** A total of 516 differential peaks were selected. GO analysis of functions revealed that promoter, untranslated region (UTR), exon, intron, and intergenic genes were mainly enriched in biological pathways such as keratinization, regulation of cAMP metabolic process, blood coagulation, fibrin clot formation, midgut development, and synapse assembly. Genes were mainly enriched in KEGG pathways including pentose phosphate pathway, glycosphingolipid biosynthesis—globo and isoglobo series, ECM–receptor interaction, and adherens junction. In total, 244 nodes and 475 interaction pairs were included in the PPI network with the hub genes including *EGFR*, *CTNNB1*, and *SPTAN1*.

**Conclusion:** EGFR, SPTAN1, and CTNN1B might be the potential down-stream genes of ASCL1 in GBM development, and CTNN1B might make contributions to GBM progression on regulating the cAMP pathway.

## Introduction

Glioblastoma (GBM), as the most common primary malignant brain tumor in adults, is one of the most aggressive and lethal human tumors characterized by a block in cellular differentiation. The median survival can range from 12 to 15 months among patients undergoing the current standard of care treatment involving surgery, chemotherapy, and radiation therapy (Ji et al., 2015; Shabihkhani et al., 2017; Xu et al., 2017; Mortazavi, 2018; Jin et al., 2019). For GBM patients, the disease is hardly diagnosed in the early stage. Meanwhile, recent therapeutic options are limited and prognosis is poor (Berninger et al., 2007). Due to the extremely high malignant grade of GBM, surgical resection combined with radiotherapy and chemotherapy has not changed its malignant progression trend, which is a serious threat to human health. Thus, novel treatment paradigms are urgently needed to improve outcomes. Currently, many efforts are focused on the target therapies, such as traditional small molecule inhibitors, monoclonal antibodies, and immunotherapeutic approaches (Park et al., 2017). These treatment strategies are actively examined in clinical trials and offer an attractive alternation (Shao et al., 2013; Narayanan et al., 2018; Bao et al., 2020). At present, various bioinformatics methods have sprung up, and a large amount of tumor gene expression profile data have become the research direction of tumor precision therapy. Therefore, the study of the glioma gene expression network also has an important theoretical value and practical significance, and its clinical application prospect should not be rested.

ASCL1 is a gene classifier for the pro-neural (PN) transcriptional subgroup of GBM, which plays as a relevant role in the neuronal-like differentiation of glioblastoma stem cells (GSCs). It has been noted that cell-cycle exit and full neuronal specification and differentiation could be induced by ASCL1 over-expression in neural precursor cells (Barrett et al., 2013). Park *et al.* demonstrated that the transcription factor ASCL1 was required for GSCs to undergo neuronal lineage differentiation, and GSCs with high ASCL1 expression levels were responsive to notch pathway inhibitors and important in driving neuronal fate (Kent et al., 2002; Langmead and Salzberg, 2012; Bolger et al., 2014). Furthermore, Narayanan proposed that ASCL1 might be served as potential subgroup-specific targetable vulnerability in GBM through targeting NDRG1 (Zhang et al., 2008; Stark, 2011; Bao et al., 2018; Ji et al., 2019; Bao et al., 2021). These efforts suggest that ASCL1 plays important roles in neuronal specification. However, the molecular network associated with the roles of ASCL1 in GBM has not yet been researched.

In order to clarify the regulatory mechanisms of the ASCL1 in GBM, the data of ChIP-seq were analyzed by utilizing bioinformatics method. The annotated gene functions and signaling pathways were investigated by Gene ontology (GO) function and kyoto encyclopedia of genes genomes (KEGG) pathway enrichment analysis. Moreover, the protein–protein interaction network (PPI) analysis of genes was constructed to explore the hub genes influenced by ASCL1.

## Materials and methods

### Data sources

GSE87618 was the genome occupancy profiling of differential ASCL1 binding between control and GSC cultures induced to overexpress ASCL1 after 18 h of doxycycline treatment, which were downloaded from the database of Gene Expression Omnibus (GEO, http://www.ncbi.nlm.nih.gov/geo/) (Robinson and Oshlack, 2010). GSE87618 contained data from eight samples, including three ASCL1 ChIP-seq negative controls (nc1, nc2, nc3), one ASCL1 ChIP-seq negative control input (nc_input), three ASCL1 ChIP-seq doxycycline (dox1, dox2, dox3, 18 h of doxycycline treatment), and one ASCL1 ChIP-seq doxycycline input (dox_input, 18 h of doxycycline treatment). Sra format profile data of GSE87618 were downloaded, and the microarray data were then converted into. fastq utilizing fastq-dump (https://trace.ncbi.nlm.nih.gov/Traces/sra/sra.cgi?view=toolkit_doc&f=fastq-dump).

### Quality control of sequencing data

In order to filter out the unreliable bases and reads, quality control was performed for the original offline data. Sequencing tape joints were firstly removed. Reads with consecutive masses below 20 at both ends or reads less than 36 nt in length would be removed. Clean reads were obtained by utilizing the tool of Trimmomatic (v3.6) (Yu et al., 2015; Bao et al., 2020).

### Sequence alignment

The filtered clean reads were mapped to the human genome (UCSC, hg19), utilizing the software of bowtie 2 (Shannon et al., 2003; Huang et al., 2009; Yu et al., 2012; Szklarczyk et al., 2015). The non-unique mapped reads and low-quality mappings in the results were removed, utilizing default parameters.

### Peak calling

Peak signal detection is a key step in the ChIP-seq analysis. MACS2 was used to find the peaks of the enrichment region of trusted sequence (the ASCL1 binding region) from the short sequence alignment results and predict the length of the predicted frag_sizes (Dablander and Hinne, 2019). The default parameters and *p*-value< 1e-3 were set as the screening threshold.

### Differential peak analysis

Based on the obtained alignment result and the peak call result, differential peak analysis was performed, utilizing diffbind software (Wang et al., 2008) to obtain differential peak binding to the chromosome due to the expression level of ASCL1 between the ASCL1 ChIP-seq doxycycline group and negative control group. The downstream analysis was performed on an overlap peak in at least three samples, and the number of reads covered by the peak was calculated to obtain the binding affinity matrix. Then, the differential peaks were calculated by edgeR (Du et al., 2015; Tang et al., 2015). The screening thresholds were designed as false-positive rate (FDR) < 0.05 and |Fold change | > 2.

### Peak annotation

The differential peaks obtained in the previous step were annotated, utilizing Chipseeker (Rheinbay et al., 2013). A 3 kb (up: 2500 bp down: 500 bp) sequence near the transcription start site (TSS) was selected as a promoter region.

### Gene ontology function and kyoto encyclopedia of genes and genomes pathway enrichment analysis

Enriched GO function and KEGG pathway were analyzed on the annotated genes (Zhang et al., 2008). The genes were divided into five categories based on different positions, including promoter, UTR, exon, intron, and intergenic. KEGG pathways and GO functions were analyzed for genes on different positions, respectively, utilizing the tool of Database for Annotation, Visualization and Integrated Discovery (DAVID) based on hypergeometric test (Azzarelli et al., 2022). The significant threshold was set as *p* value <0.05.

### Protein–protein interaction network

The database of Search Tool for Retrieval of Interacting Genes (STRING) is an online database for predicting PPIs (Wang et al., 2021). Utilizing STRING (version 10.0, http://www.string-db.org/) database, the PPIs of genes were analyzed. The protein pairs with PPI score >0.4 were collected. Then, Cytoscape (version: 3.2.0, http://www.cytoscape.org/) was used to visualize the predicted PPI network (Gorla et al., 2009; Bhinge et al., 2017; Vue et al., 2020).

Three methods were used to evaluate the centrality of the complex network, including degree centrality (Ackermann et al., 2019), betweenness centrality (Nager et al., 2018), and closeness centrality (Chen et al., 1998). CytoNCA was a cytoscape plugin for the calculation of three topology properties (parameter setting: network is without weight) (Mahesparan et al., 1997; Woods et al., 2022). In the CytoNCA output, the node score represented the role of the protein in the network.

## Results

### Sequencing data quality control and sequence data comparison result

Table 1 shows the quality of sequencing data. The percentages of bases in all eight samples with Phred values greater than 30 were all more than 99.8%. The results of sequencing data comparison are shown in Table 2, and the mapped rates in different samples were all more than 95%.

**TABLE 1:** Quality of sequencing output data.

Sample	raw_reads	base_base	clean_read	clean_base	clean_read_ QC30 (%)	Gc% (%)
dox1	30270652	3057335852	27858530	2783226852	99.88	42
dox2	28593135	2887906635	26234044	2621375448	99.88	42
dox3	26781109	2704892009	24625563	2461120368	99.89	42
dox_input	33156476	3348804076	31951206	3201740411	99.92	42
nc1	29060175	2935077675	26104377	2609039566	99.88	42
nc2	27823338	2810157138	25390202	2537614866	99.88	42
nc3	28106737	2838780437	25336418	2533215072	99.90	42
nc_input	32015101	3233525201	30780580	3081984505	99.92	43

**TABLE 2:** Comparison results of sequencing data.

Sample	clean_read	Mapped	Mapped rate (%)	Unique mapped	Unique mapped rate (%)
dox1	27858530	26736498	95.97	23031768	82.67
dox2	26234044	25089241	95.64	21628215	82.44
dox3	24625563	23615202	95.90	20451291	83.05
dox_input	31951206	30733109	96.19	26421023	82.69
nc1	26104377	24943337	95.55	21355241	81.81
nc2	25390202	24294770	95.69	20820887	82.00
nc3	25336418	24320000	95.99	21004213	82.90
nc_input	30780580	29595109	96.15	25423607	82.60

### Peak call based on the expression level of ASCL1

In total, 4792 peaks were obtained in sample DOX_1, 4176 peaks in DOX_2, 4231 peaks in DOX_3, 1211 peaks in NC_1, 1193 peaks in NC_2, and 733 peaks in NC_3. The doxycycline treatment resulted in a higher expression of ASCL1, and then more peaks were obtained.

### Differential peak analysis

**FIGURE 1:**
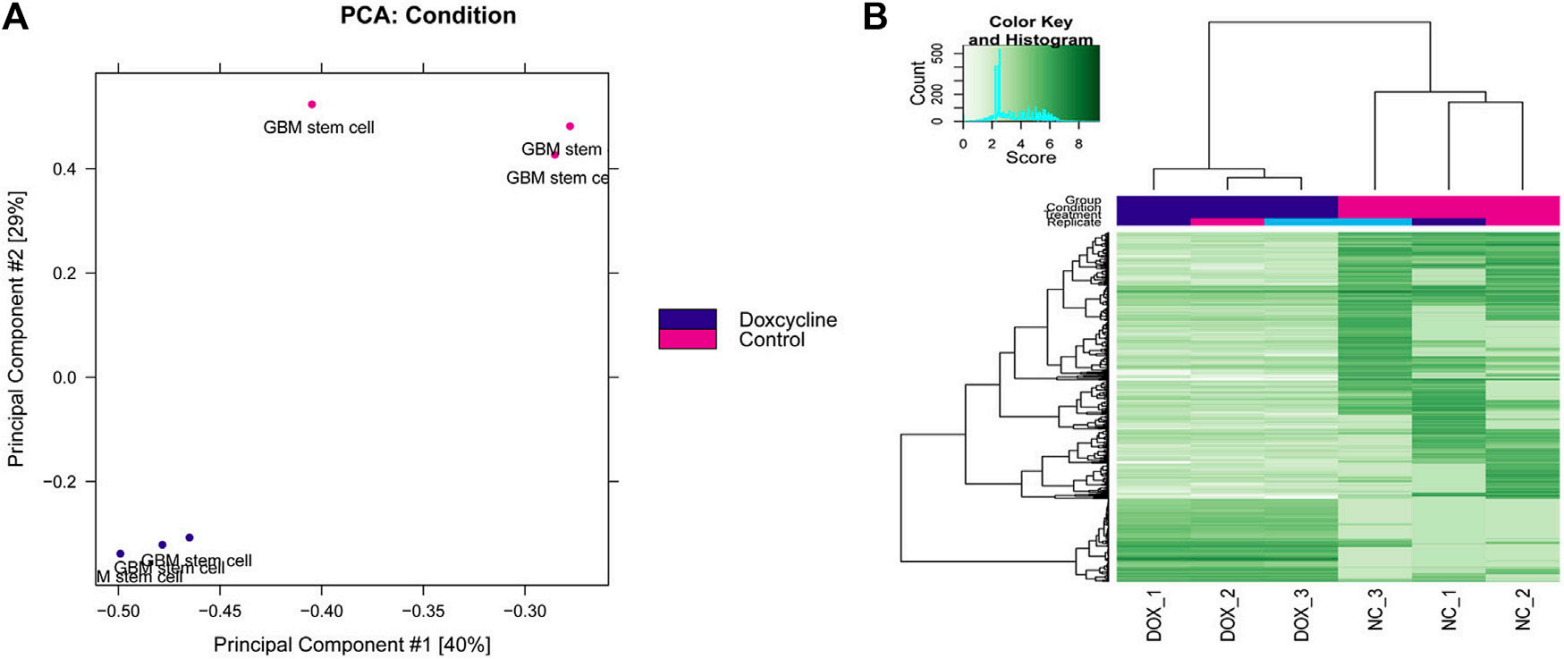
Principal component analysis **(A)** and two-dimensional hotspot clustering map of differential peaks **(B)**.

The differential peak analysis was performed, and a total of 516 differential peaks were selected. The results of the principal component analysis (PCA) between multiple samples are shown in Figure 1A, and the samples in control and doxycycline treatment group were clustered separately. Differential expression peak hotspot distribution map showed that the profile of differential expression peaks was significantly different in the control and doxycycline treatment group (Figure 1B).

### Differential peak annotation

**FIGURE 2:**
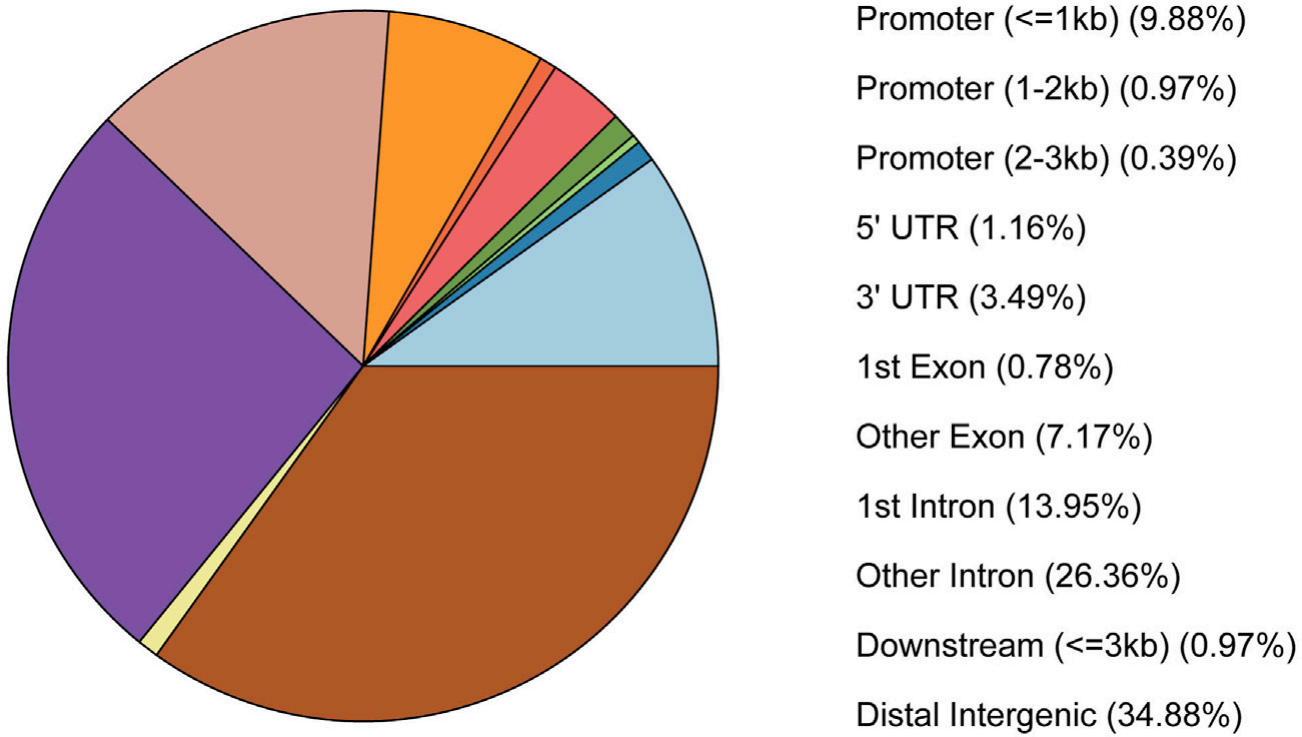
The pie chart of peaks annotated into the genomic region.

Peaks were annotated by peakseeker, and results showed that 10% peaks were located near the gene promoter, 33% of the peaks were located in the intergenic region, and 40% of the peaks were located in the intron region of the genes (Figure 2).

### GO function and KEGG pathway enrichment of genes involved in differential peak annotation

**FIGURE 3:**
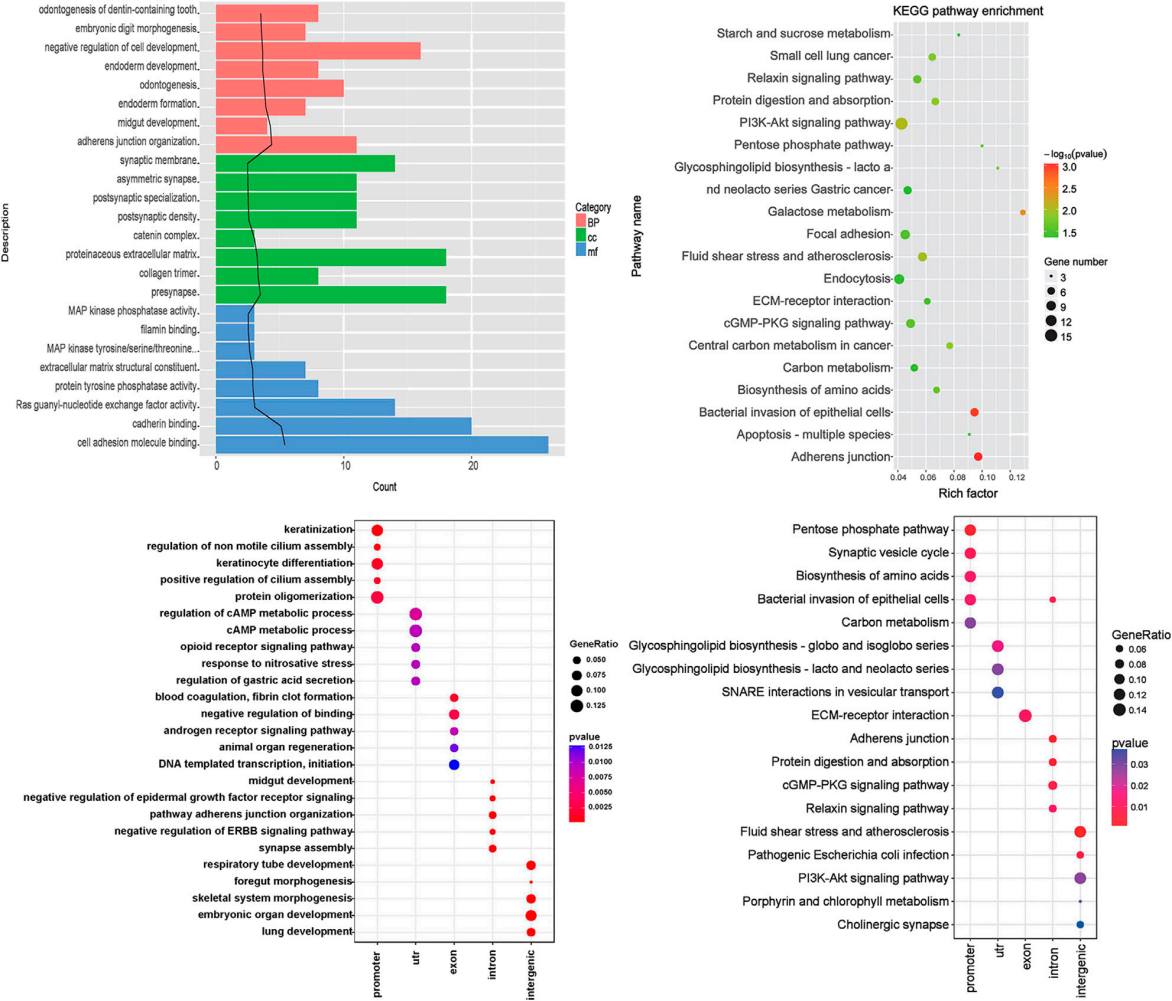
Functional enrichment analysis. **(A)** Gene ontology (GO) enrichment analysis. Category: the category of GO, BP: biological pathway, CC: cell composition, MF: molecular function, term: GO function description information, count: the number of differential genes enriched in the term; the black trend line represents the -log10 (*p* Value) value. **(B)** The KEGG pathway enrichment analysis results of differential genes. The rich factor refers to the ratio of the number of differentially expressed genes in the pathway to the total number of genes in the pathway. The size of the rich factor represents the degree of enrichment. **(C)** Enriched biological pathway of five kinds of genes; **(D)** KEGG pathway of five kinds of genes. The abscissa represents the group name, and the ordinate represents the enrichment entry name.

According to the results of the differential peak annotation, the obtained genes were separately subjected to GO functional and KEGG pathway enrichment analysis. As shown in Figure 3A, genes are mainly enriched in biological pathways, such as “odontogenesis of dentin-containing tooth,” “embryonic digit morphogenesis,” and “negative regulation of cell development.” Figure 3B shows that genes are mainly enriched in KEGG pathways, including “starch and sucrose metabolism,” “small cell lung cancer,” and “relaxin signaling pathway.”

Genes were divided into five categories including promoter, UTR, exon, intron, and intergenic. Functional enrichment analysis was further performed, and the results showed that promoter genes were mainly enriched in biological pathways such as keratinization. UTR genes were enriched in the regulation of the cAMP metabolic process. Exon genes were enriched in blood coagulation and fibrin clot formation, intron genes were enriched in midgut development, and intergenic genes were enriched in synapse assembly (Figure 3C). KEGG pathway analysis showed that the significant pathways included pentose phosphate pathway (promoter genes), glycosphingolipid biosynthesis—globo and isoglobo series (UTR genes), ECM–receptor interaction (exon genes), adherens junction (intron genes), and fluid shear stress and atherosclerosis (intergenic genes) (Figure 3D).

### PPI network

**FIGURE 4:**
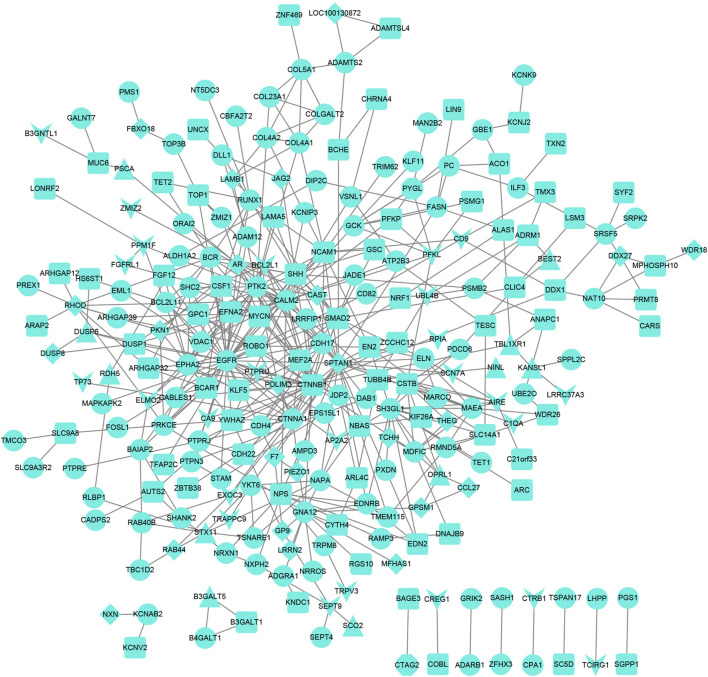
Protein–protein interaction network. The V-shaped node represents the promoter, the triangular node represents the UTR, the diamond node represents the exon, the circular node represents the intron, and the square node represents the intergenic region.

The PPI network for genes was constructed (Figure 4), and 244 nodes and 475 interaction pairs were included in the network. The top 10 hub nodes based on the degree centrality, betweenness centrality, and closeness centrality are shown in Table 3, such as epidermal growth factor receptor (*EGFR*), catenin Beta 1 (*CTNNB1*), and spectrin alpha, non-erythrocytic 1 (*SPTAN1*).

**TABLE 3:** The top 10 hub nodes in the protein–protein interaction network.

name	Degree	name	Betweenness	name	Colseness
EGFR	41	CTNNB1	13460.64851	CTNNB1	0.041263
CTNNB1	33	CALM2	11008.59049	EGFR	0.041179
SPTAN1	27	SPTAN1	10851.35589	SPTAN1	0.041117
CALM2	27	EGFR	10807.95974	CALM2	0.041117
PTK2	17	GNA12	4305.269611	PTK2	0.040772
SH3GL1	17	DDX1	3878.215375	AR	0.040772
SHH	16	PTK2	3480.394513	SHH	0.040669
CTNNA1	15	AR	3439.149015	BCL2L1	0.040649
AR	14	SHH	3354.04941	MYCN	0.040602
NCAM1	13	SH3GL1	2918.910619	CTNNA1	0.040595
GNA12	13	NPS	2559.36073	CDH17	0.04048
EPHA2	12	NCAM1	2169.773138	YWHAZ	0.040412
BCL2L1	10	TBL1XR1	2112.331093	NCAM1	0.040406
SMAD2	10	NAT10	1869.07619	SMAD2	0.040352
MYCN	10	FASN	1813.87283	MEF2A	0.040319

## Discussion

GBMs are incurable brain tumors with a high degree of cellular heterogeneity and genetic mutations. For anti-GBM therapies, the loss of proliferation control and unregulated self-renewal would be the most important hard work for clinical treatment. In the context of gliomas, some transcription factors are often expressed and have been shown to function in determining the tumorigenicity and differentiation status of tumor cells. In this study, we focus on ASCL1, a class II basic-helix–loop–helix (bHLH) transcription factor that forms a heterodimer with class I bHLH E-proteins (such as E47/TCF3) to activate specific target genes. Studies have shown that ASCL1 regulates transcriptional targets that are central to the GBM development. The study of Park *et al.* showed that ASCL1 could control the neuronal fate and suppress tumorigenicity of glioblastoma stem cells by reorganizing chromatin (Park et al., 2017). The study of Rheinbay *et al.* showed that ASCL1 exerts functions in part by repressing an inhibitor of Wnt signaling, DKK1, resulting in increased signaling through this pathway to maintain the tumorigenicity of glioma cells (Shannon et al., 2003). ASCL1 phosphorylation and ID2 upregulation are roadblocks to glioblastoma stem cell differentiation (Wang et al., 2008; Dablander and Hinne, 2019). The study of Tou *et al.* showed that the loss of ASCL1 significantly reduces the proliferation of GBMs induced in the brain of a genetically relevant glioma mouse model, resulting in extended survival times, illustrating an important role for ASCL1 in controlling the proliferation of GBM (Du et al., 2015). In our study, a total of 516 differential peaks between ASCL1 overexpressed GSC samples and negative controls were screened. GO analysis of functions revealed that the genes annotated on differential peaks were mainly associated with biological pathways such as keratinization, regulation of cAMP metabolic process, blood coagulation, fibrin clot formation, midgut development, and synapse assembly. Genes were mainly enriched in KEGG pathways including pentose phosphate pathway, glycosphingolipid biosynthesis—globo and isoglobo series, ECM–receptor interaction, adherens junction, and fluid shear stress and atherosclerosis. The PPI network with 244 nodes and 475 interaction pairs was constructed including the hub genes such as *EGFR*, *CTNNB1*, and *SPTAN1*.

The gene expression was mediated by ASCL1 binding to chromatin. In particular, a member of the basic helix–loop–helix (BHLH) family of transcription factors was encoded by ASCL1, and the protein was important in the neuronal differentiation, olfactory, and autonomic neuron generation. In our study, a regulation network associated with ASCL1 was constructed based on ChIP-seq data. In PPI network, *EGFR*, *CTNNB1*, and *SPTAN1* were hub genes. In lung adenocarcinomas patients, EGFR mediates the activation of RET with neuroendocrine differentiation characterized by ASCL1 expression, implicating that EGFR is a key regulator of RET (Tang et al., 2015). ASCL1 function is an upstream regulator of the Ret Proto-Oncogene, so combined with our findings, we speculate that in GBM, ASCL1 may mediate RET activation through EGFR, thereby affecting tumor progression. The protein encoded by *SPTAN1* has been implicated in DNA repair and cell cycle regulation. Meanwhile, this gene was involved with the RET signaling pathway (Rheinbay et al., 2013). Ackermann *et al.* demonstrated a close relationship between low SPTAN1 expression and tumor progression and metastasis in colorectal cancers (Azzarelli et al., 2022). Although no direct evidence has shown the association between ASCL1 and the genes such as EGFR and SPTAN1 in GBM, the data revealed its potential value as an important downstream gene of ASCL1 in GBM.

It has been reported that WNT-CTNN1B signaling plays important roles in promoting cancer cell proliferation and stemness, and Nager *et al.* showed that silencing CTNN1B could decrease cell viability and induce GBM cell apoptosis (Wang et al., 2021). In malignant gliomas, previous evidence showed that protein kinase (PKA) activation was correlated with decreased proliferation, increased differentiation, and apoptosis induction by increasing cAMP levels or directly by cAMP analogues (Vue et al., 2020). Regulation of the cAMP metabolic process was involved in the GO enrichment function in GBM. Together with the data presented herein, there is a growing body of evidence suggesting a role of CTNN1B in GBM progression based on regulating the cAMP pathway. An uncontrolled cell proliferation and infiltrative growth within the brain were the main characteristics in malignant human gliomas. The conjunction with vascular elements has specific interactions between tumor cell surface receptors and specific ECM, which induced an extensive tumor cell movement along blood vessels (Bhinge et al., 2017). Enhancing ASCL1 activity in a neurogenic environment both increases binding at endogenous ASCL1 sites and also results in additional binding to new low affinity sites that favors neuronal differentiation (Gorla et al., 2009). Our data showed that the ECM–receptor interaction and pentose phosphate pathways were dysregulated by ASCL1 overexpression in GSCs.

Our data provide a genome-wide view of gene regulation by ASCL1 signaling in GBM, and we showed important roles of hub genes influenced by ASCL1. However, there are some limitations that should be noted. The data were all analyzed by the method of bioinformatics, and the potential functional enrichment of genes should be further researched by clinical research. Furthermore, only three ASCL1 ChIP-seq negative controls and three ASCL1 ChIP-seq doxycyclines were enrolled in the analysis. It should not be denied that the background of GBM patients varied from each other. Thus, the conclusion should be verified by further systematical analysis.

In summary, we constructed a regulation network for the ASCL1 role involved in neurogenic gene expression program activation in GBM. Our data revealed that EGFR and SPTAN1 were the potential downstream genes of ASCL1 in the GBM development, and CTNN1B might take part in GBM progression based on regulating the cAMP pathway. However, the conclusion should be further verified by experimental data.

## Data Availability

The original contributions presented in the study are included in the article/supplementary material; further inquiries can be directed to the corresponding author.

## Abbreviations

ASCL1, achaete-scute homolog 1; BHLH, basic helix–loop–helix; CTNNB1, catenin beta 1; CCHS, congenital central hypoventilation syndrome; CSCs, cancer stem cells; GBMs, glioblastomas; GO, gene ontology; KEGG, kyoto encyclopedia of genes genomes; PPI, protein–protein interaction network; PN, proneural; GSCs, glioblastoma stem cells; FDR, false-positive rate; PCA, principal component analysis; EGFR, epidermal growth factor receptor; SPTAN1, spectrin alpha, non-erythrocytic 1.
